# Prolonged Induction Activates *Cebpα* Independent Adipogenesis in NIH/3T3 Cells

**DOI:** 10.1371/journal.pone.0051459

**Published:** 2013-01-10

**Authors:** Hsiao-Yun Shao, Hsue-Yin Hsu, Kuan-Sju Wu, Siow-Wey Hee, Lee-Ming Chuang, Jih-I Yeh

**Affiliations:** 1 Graduate Institute of Molecular and Cell Biology, Tzu Chi University, Hualien, Taiwan; 2 Department and Graduate Institute of Life Sciences, Tzu Chi University, Hualien, Taiwan; 3 Institute of Medical Sciences, Tzu Chi University, Hualien, Taiwan; 4 Institute of Molecular Medicine, National Taiwan University, Taipei, Taiwan; 5 Department of Family Medicine, Tzu Chi University, Hualien, Taiwan; 6 Department of Family Medicine, Buddhist Tzu Chi General Hospital, Hualien, Taiwan; Brigham and Women's Hospital, United States of America

## Abstract

**Background:**

3T3-L1 cells are widely used to study adipogenesis and insulin response. Their adipogenic potential decreases with time in the culture. Expressing exogenous genes in 3T3-L1 cells can be challenging. This work tries to establish and characterize an alternative model of cultured adipocytes that is easier to work with than the 3T3-L1 cells.

**Methodology/Principal Findings:**

Induced cells were identified as adipocytes based on the following three characteristics: (1) Accumulation of triglyceride droplets as demonstrated by oil red O stain. (2) Transport rate of 2-deoxyglucose increased after insulin stimulation. (3) Expression of fat specific genes such as Fabp4 (aP2), Slc2a4 (Glut4) and Pparg (PPARγ). Among the cell lines induced under different conditions in this study, only NIH/3T3 cells differentiated into adipocytes after prolonged incubation in 3T3-L1 induction medium containing 20% instead of 10% fetal bovine serum. Rosiglitazone added to the induction medium shortened the incubation period from 14 to 7 days. The PI3K/AKT pathway showed similar changes upon insulin stimulation in these two adipocytes. C/EBPα mRNA was barely detectable in NIH/3T3 adipocytes. NIH/3T3 adipocytes induced in the presence of rosiglitazone showed higher 2-deoxyglucose transport rate after insulin stimulation, expressed less Agt (angiotensinogen) and more PPARγ. Knockdown of C/EBPα using shRNA blocked 3T3-L1 but not NIH/3T3 cell differentiation. Mouse adipose tissues from various anatomical locations showed comparable levels of C/EBPα mRNA.

**Conclusions/Significance:**

NIH/3T3 cells were capable of differentiating into adipocytes without genetic engineering. They were an adipocyte model that did not require the reciprocal activation between C/EBPα and PPARγ to differentiate. Future studies in the C/EBPα independent pathways leading to insulin responsiveness may reveal new targets to diabetes treatment.

## Introduction

Much has been learned about the molecular basis of adipocyte formation and insulin action using cell culture models of adipocytes. These models of adipocytes show the accumulation of triglyceride droplets, express adipocyte marker genes and increase glucose uptake in response to insulin stimulation [1]. One of the most widely used cell culture models of adipocytes is 3T3-L1 cells. Confluent 3T3-L1 cells form insulin responsive adipocytes spontaneously in 2–4 weeks [2], [3]. Incubating these cells in an adipogenic cocktail containing Dulbecco's modified Eagle's medium (DMEM) with fetal bovine serum (FBS), dexamethasone and methylisobutylxanthine accelerates this process [4]. The conditions for 3T3-L1 adipocyte induction have been used in other cell types to assess their adipogenic potential. Multipotential fibroblasts NIH/3T3 cells [5] were used as negative controls in many experiments because they could not form adipocytes when induced like 3T3-L1 cells [6].

Rosen et al synthesized all available evidence and proposed that the reciprocal activation between Cebp*a* (C/EBPα) [7] and Pparg (PPARγ) was required to turn on the program of adipogenesis in all models of adipocytes [8]. These two genes were also critical to insulin response in adipocytes. They were always expressed together but not functionally equivalent in adipocytes. Over-expression of either C/EBPα or PPARγ in NIH/3T3 cells enabled them to accumulate fat droplets. The cells derived from C/EBPα over expressing NIH/3T3 cells were insulin responsive and expressed PPARγ while those derived from PPARγ overexpressing cells were not insulin responsive and did not express C/EBPα. These results lead to the belief that C/EBPα was required for insulin response and acted upstream of PPARγ which was the master regulator of the morphological transformation to adipocytes [6].

The use of 3T3-L1 cells was sometimes hampered by the gradual loss of their adipogenic potential in the culture over time and their resistance to gene transfer and expression. Adding PPARγ agonists, such as troglitazone or rosiglitazone [9], [10] to the induction medium or extending induction time to 3 or 4 days to compensate for the loss of adipogenic potential in the ageing cells were quite common in the literature. These measures and the adipogenic induction cocktail only accelerated but were otherwise not essential in 3T3-L1 differentiation. The practice to boost the adipogenicity of ageing 3T3-L1 cells by inducing them for more than 3 days suggested that some adipogenic cells required longer induction time to show their adipogenic potential. We suspected that some cells that could not form adipocytes when induced like 3T3-L1 cells might have formed adipocytes if given longer induction time. This work tried to find new models of adipocytes by screening and characterizing cell lines that could not form adipocytes when induced like 3T3-L1 cells but could do so when given longer induction time.

## Results

The 3T3-L1 cells have been used for more than 3 decades. They were always induced after they became confluent so that contact inhibition was considered a prerequisite for adipogenesis [11]. Since the declining differentiation potential of ageing 3T3-L1 cells in the culture could often be compensated by longer induction period with the adipogenic cocktail, we suspected that lack of contact inhibition could also be compensated by longer induction period. 3T3-L1 preadipocytes were incubated in the adipogenic cocktail (10% FBS in DMEM with 1 µM dexamethasone and 0.5 mM methylisobutylxanthine) when they were about 20–30% confluent. Few cells accumulated fat droplets if induced with the cocktail for only 3 days. Increase this period to 6 days significantly improved the differentiation (data not shown). The cell number also increased during this period but did not became confluent. The induction time could be shortened with the PPARγ agonist rosiglitazone to 3 days. These adipocytes were equaly insulin responsive in 2-deoxyglucose uptake as regular 3T3-L1 adipocytes (data not).

**Figure 1:**
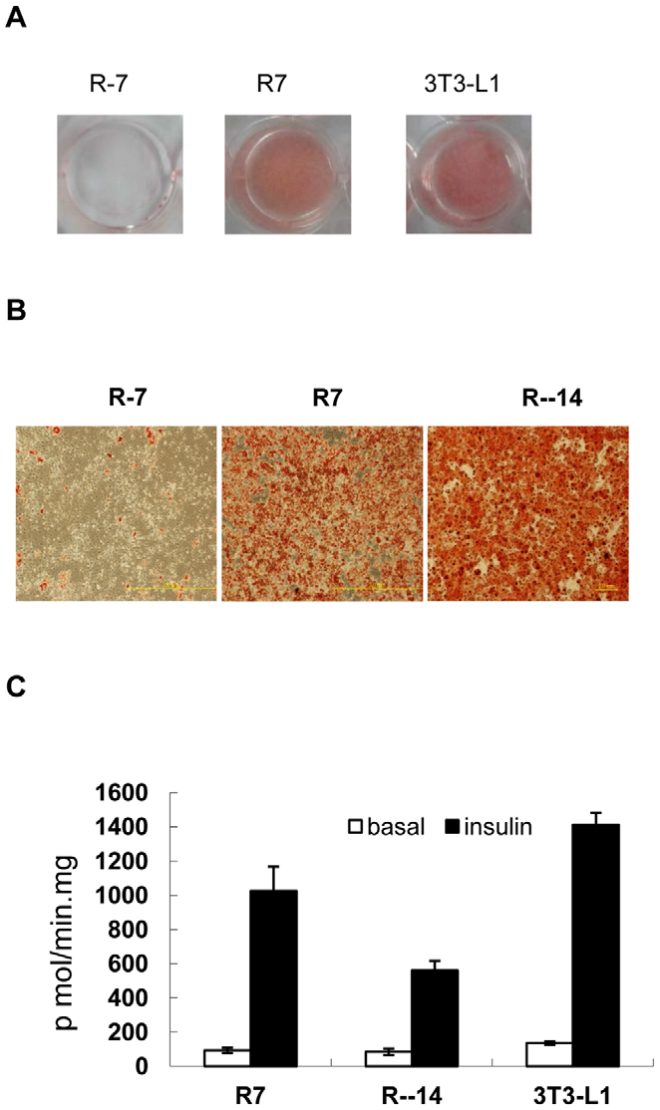
NIH/3T3 cells formed insulin responsive adipocytes after prolonged induction. (A) The whole cell culture dish view of the oil red O stain of NIH/3T3 cells induced with (R7) or without rosiglitazone (R-7) for 7 days and regular 3T3-L1 (3T3-L1) adipocytes. (B) Oil red O stained images of NIH/3T3 cells induced without rosiglitazone for 7 (R-7) or 14 days (R-14) and with rosiglitazone for 7 days (R7). (C) The glucose uptake rate in response to insulin with the standard errors (n = 3) of measurement of cells in panel (B).

**Figure 2:**
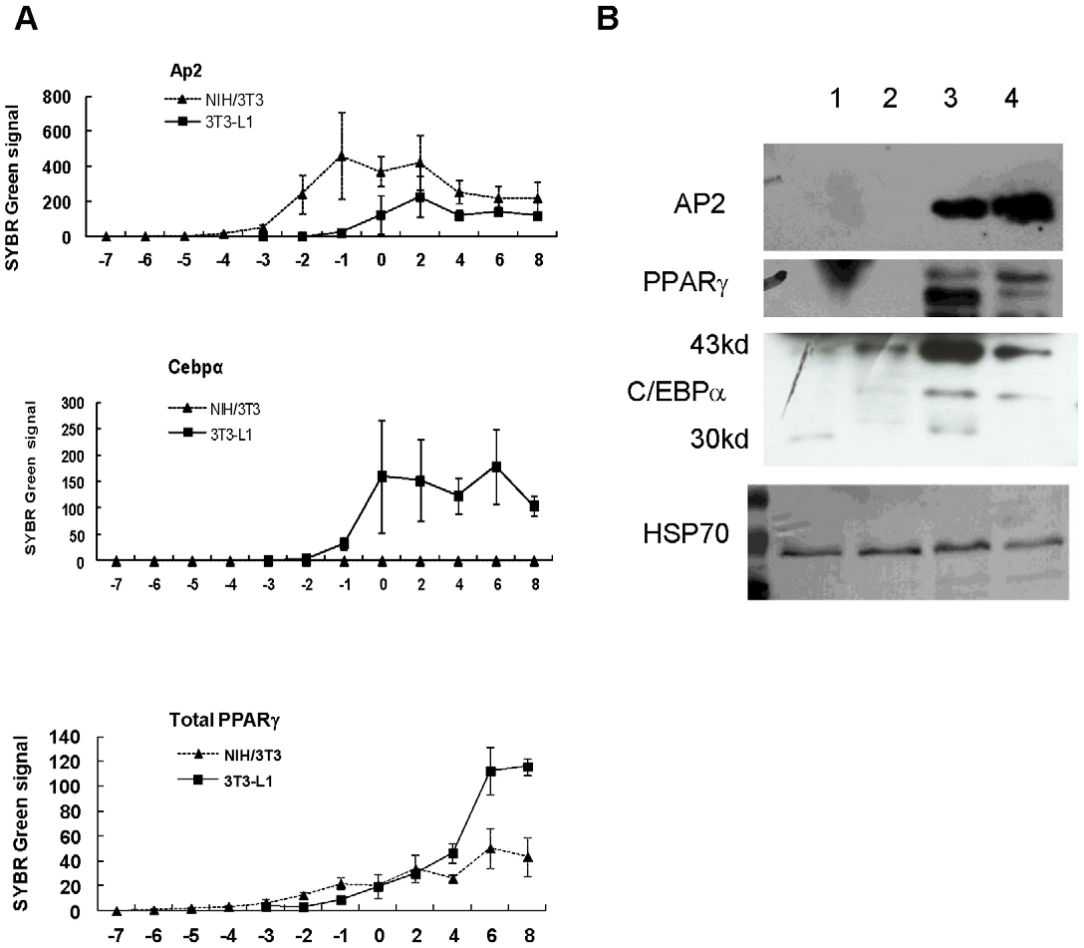
NIH/3T3 adipocytes did not express C/EBPα. The relative mRNA levels and the standard errors (n = 3) of measurement of 3 adipocyte marker genes, (A) Fabp4 (Ap2), (B) Cebpa (C/EBPα) and (C) Pparg (PPARγ) were determined daily by qPCR during adipogenesis in 3T3-L1 and NIH/3T3 (R7) cells. Because the required induction times were different in these 2 cell lines, day 0 on the x-axis indicated the day the induction medium was removed and the cells were incubated in maturation medium. (D) Western blot of labeled genes in 3T3-L1 and NIH/3T3 fibroblasts and adipocytes. HSP70 was used as the internal control. Lanes 1: 3T3-L1fibroblasts, 2: NIH/3T3 fibroblasts, Lanes 3: 3T3-L1adipocytes, 4: NIH/3T3 adipocytes.

**Figure 3:**
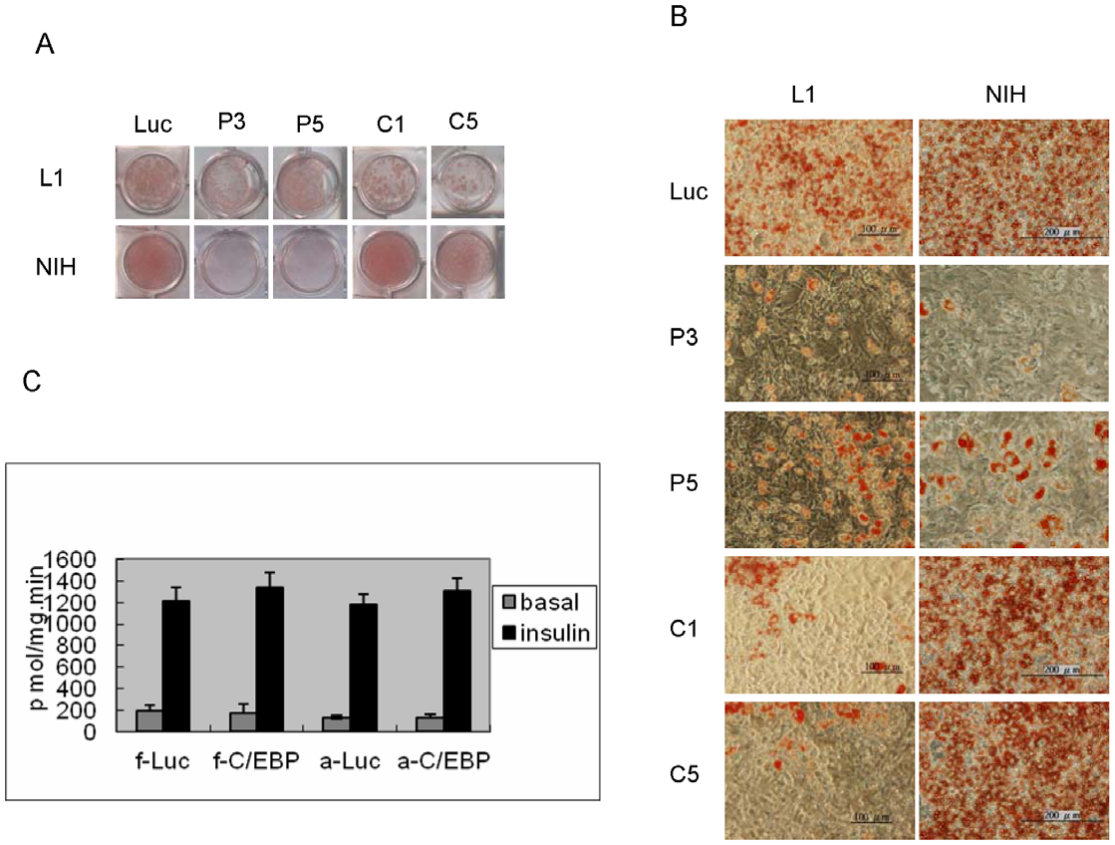
C/EBPα was not required for adipogenesis in NIH/3T3 cells. (A) The whole cell culture dish view in panel (A) and micrographs in panel (B) of the oil red O staining of 3T3-L1 (L1) and NIH/3T3 (NIH) cells induced after the cells were infected with lentiviruses expressing shRNA targeting luciferase (Luc), C/EBPα (C1 and C5) or PPARγ (P3 and P5). (C) The 2-deoxyglucose uptake and the standard errors (n = 3) of measurement of NIH/3T3 adipocytes infected with lentiviruses expressing shRNA targeting luciferase (-Luc) or C/EBPα (-C/EBP) before (f-) or after (a-) differentiation of NIH/3T3 cells.

The effect of longer induction time to increase adipogenesis was then tested on several murine cell lines, including NIH/3T3, CT26 [12], KLN 205 [13], mouse embryonic fibroblasts. NIH/3T3 cells were the only cell line that showed significant differentiation when induced in the 3T3-L1 adipogenic cocktail for 2 weeks in the absence of rosiglitazone. Less than 40% of cells accumulated fat droplets. When the FBS in the adipogenic cocktail was increased to 20%, more than 90% of the cells differentiated. The induction time could be decreased to 1 week if 4.5 µM rosiglitazone was added (Figures 1A and B). The insulin stimulated 2-deoxyglucose uptake increase in NIH/3T3 adipocytes induced with rosiglitazone was about twice of that in NIH/3T3 adipocytes induced without rosiglitazone (Figure 1C). NIH/3T3 cells were well known for their inability to express C/EBPα even in the presence of exogenous PPARγ [6]. Given the central role of C/EBPα in adipogenesis and insulin response in the molecular model of adipogenesis, this pattern of transcription regulation would be incompatible with the formation of insulin responsive adipocytes. To address this issue, the mRNA levels of adipocyte markers, including C/EBPα, PPARγ, Slc2a4 (Glut4) and Fabp4 (aP2) were compared in 3T3-L1 and NIH/3T3 cells before and after differentiation by semi quantitative PCR. Only C/EBPα showed significant difference between these adipocytes (data not shown). The kinetics of aP2, C/EBPα and PPARγ mRNA induction during adipogenesis was assessed by quantitative PCR (Figures 2A). The NIH/3T3 cells had barely detectable C/EBPα mRNA level throughout the differentiation process. The 43kd isoform of C/EBPα in NIH/3T3 adipocytes was comparable to that in 3T3-L1 fibroblasts and the 30kd isoform was not detected in western blot analysis (Figure 2B). Though C/EBPα mRNA increase was barely detectable during adipogenesis of NIH/3T3, the possibility that a brief surge of its expression was essential could not be ruled out. Because of the interdependence between C/EBPα and PPARγ, the lack of detectable C/EBPα also cast doubt on the essential role of PPARγ. The roles of both genes in adipocytes formation were tested by knockdown in 3T3-L1 and NIH/3T3 cells. PPARγ knockdown blocked adipogenesis in both cell lines. While C/EBPα knockdown blocked adipogenesis of 3T3-L1, the same treatment had no effect in NIH/3T3 cells (Figure 3A and B). These NIH/3T3 adipocytes had the same insulin stimulated 2-deoxyglucose uptake as the controls. Expressing C/EBPα shRNA after NIH/3T3 adipocytes differentiation showed the same result. (Figure 3C). Because of low C/EBPαexpression level and the differentiation dependent expression of C/EBPα and PPARγ, the extents of their knockdown were not estimated.

**Figure 4:**
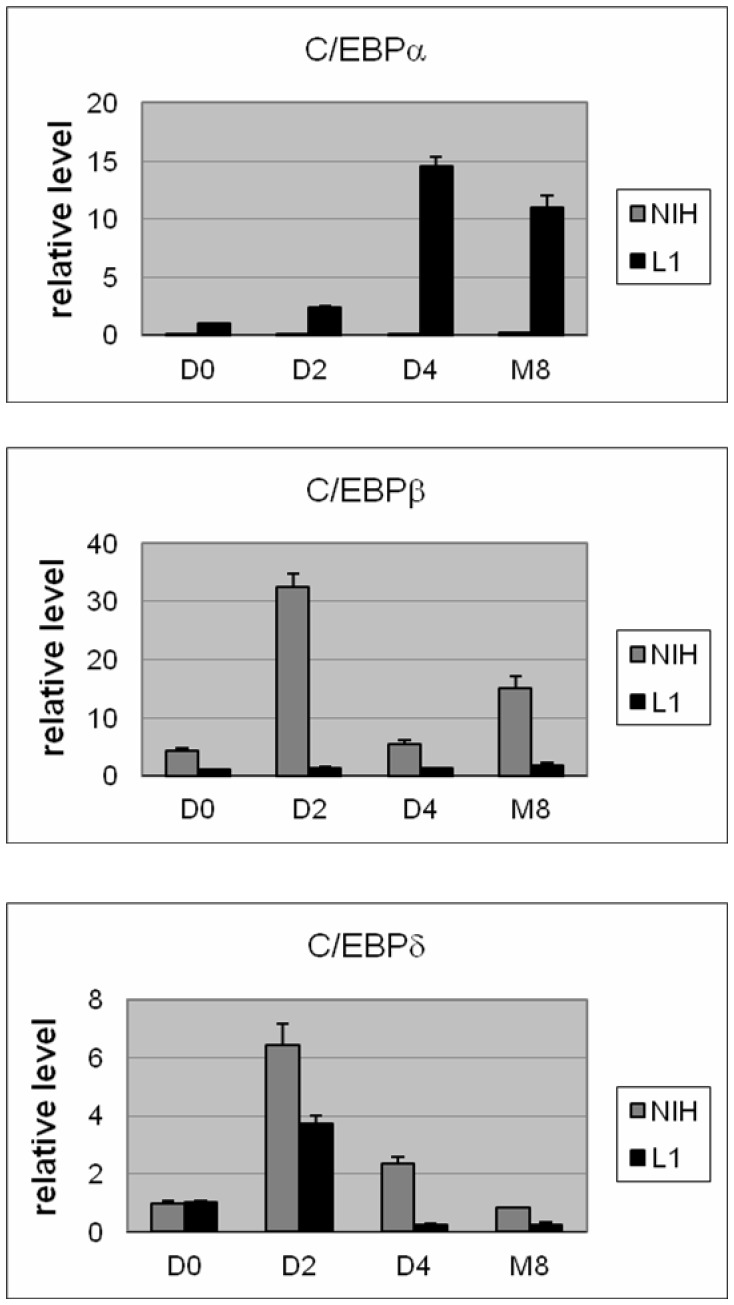
Temporal expression profiles of the C/EBPs during differentiation of 3T3-L1 and NIH/3T3 cells. The mRNA levels and the standard errors (n = 3) of measurement of (A) C/EBPα, (B) C/EBPβ, and (C) C/EBPδ were determined by qPCR and normalized by Gapdh signal on day 0 (D0), 2 (D2) and 4(D4) in differentiation medium and day 8 in DMEM +10% FBS medium.

C/EBPα encoded a member of the bZIP transcription factor family and it could form heterodimers with C/EBPβ and C/EBPδ. During 3T3-L1 adipogenesis, C/EBPβ and C/EBPδ transcription was increased earlier than C/EBPα and activated its transcription. It was possible that C/EBPβ and C/EBPδ might be increased to compensate for the lack of C/EBPα to form NIH/3T3 adipocytes. The mRNA levels of these genes were determined by qPCR before and after differentiation (Figure 4). While the C/EBPα mRNA rose and then remained near peak level throughout the differentiation process of the 3T3-L1 cells, the level of C/EBPβ and C/EBPδ mRNA peaked early in the induction phase and fell off in the NIH/3T3 cells. The relatively more abundant C/EBPβ suggested that it could be compensating for the lack of C/EBPα in NIH/3T3 adipocytes. This possibility could not be ruled out simply by the knockdown of individual or combinations of the C/EBPs because it had already been shown that C/EBPβ was required in 3T3-L1 differentiation. Inhibiting C/EBPβ activity by either expressing a dominant-negative C/EBPβ mutant [14] or C/EBPβ knockdown [15] blocked adipogenesis in 3T3-L1 cells.

**Figure 5:**
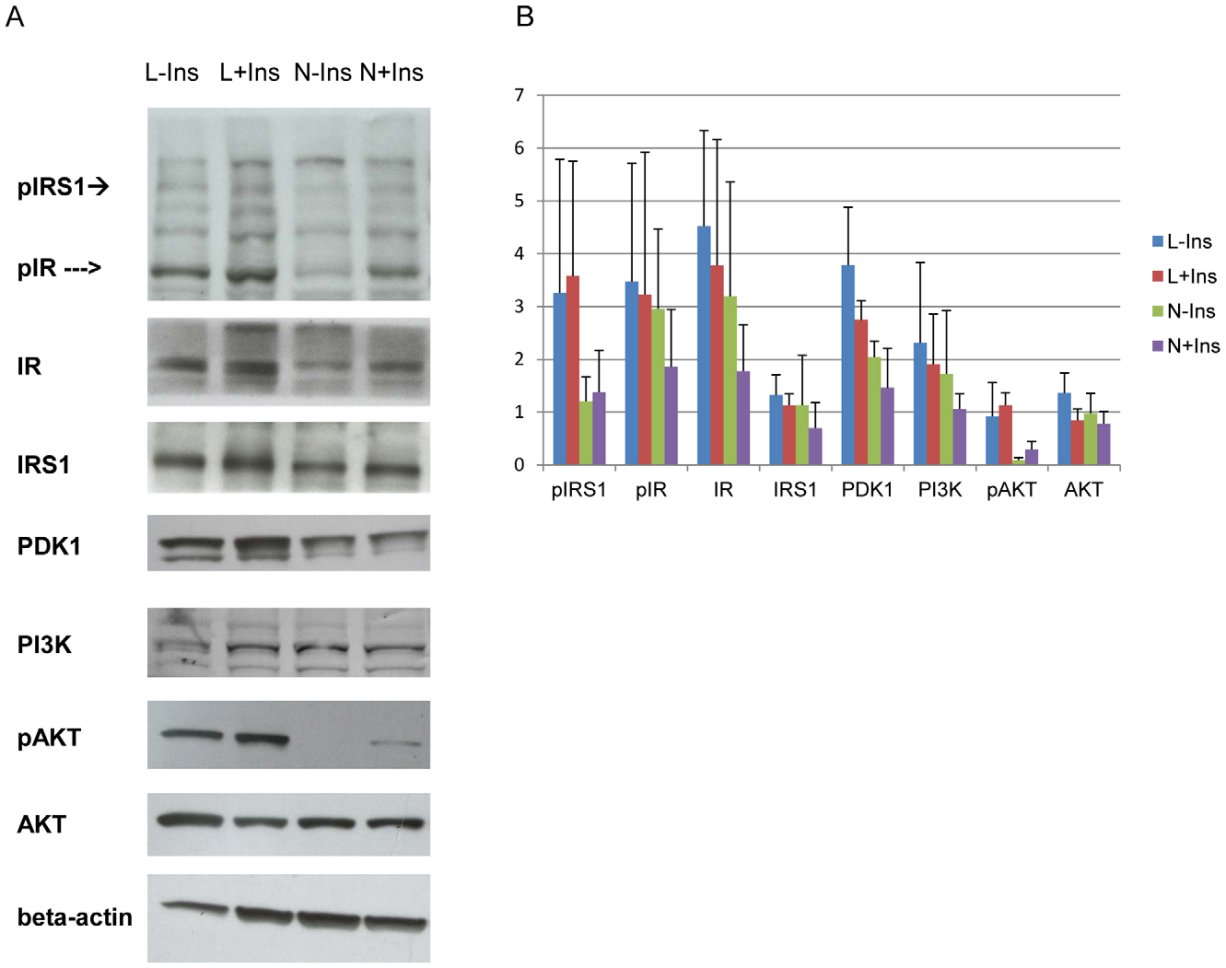
Comparison of PI3K/AKT signaling in NIH/3T3 and 3T3-L1 adipocytes. (A) Western blotting of phosphorylated-IRS-1(pIRS-1) and phosphorylated-insulin receptor (pIR), insulin receptor (IR), IRS-1, PDK1, PI3K,phospho-AKT (pAKT), total AKT (AKT), and β-actin in NIH/3T3 and 3T3-L1 adipocytes treated with (for 15 minutes) or without insulin. β-actin was the internal loading control. Labels were: L-Ins : 3T3-L1 -insulin, L+Ins: 3T3-L1 +insulin, N-Ins: NIH/3T3 -insulin, N+Ins: NIH/3T3 +insulin. pIRS-1 and pIR were blotted with 4G10 antibody, the rest of the proteins were probed with the antibody against the labeled protein. The blots were representative of 3 independent experiments. (B) The average band intensity normalized to beta-actin and the corresponding standard errors (error bars) were calculated after densitometry determination of each band in 3 independent blots as described in (A). None of the bands showed statistical significant difference in intensity.

In addition to the difference in C/EBPα expression, the PI3K/AKT signaling pathway in these two adipocytes were compared by western blotting. NIH/3T3 adipocytes showed AKT phosphorylation upon insulin stimulation as in 3T3-L1 adipocytes (Figure 5). The data in Figure 5 B show that neither AKT nor pAKT were altered by insulin treatment. This was true in both NIH/3T3 cells (with vs. without insulin) and in 3T3/L1 cells (with vs. without insulin).

**Figure 6:**
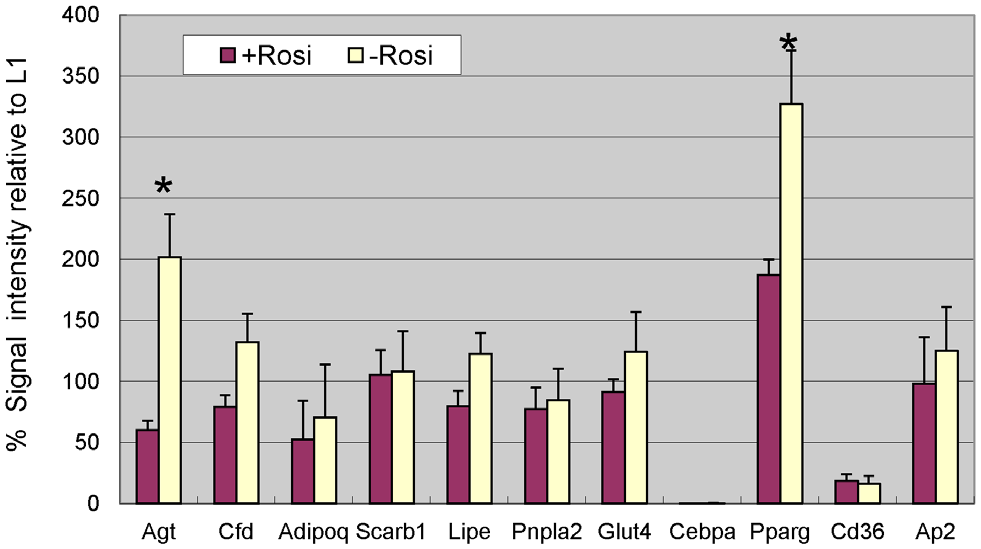
NIH/3T3 adipocytes induced without rosiglitazone expressed more *Pparg* (PPARγ) and *Agt* (Angiotensiongen). The mRNA levels and the standard errors (n = 3) of measurement of selected genes involved in blood pressure regulation, glucose transport and lipid metabolism in NIH/3T3 adipocytes induced in the presence (+Rosi) or absence (-Rosi) of rosiglitazone were compared with 3T3-L1 adipocytes. The gene symbols used were those approved by the HUGO gene nomenclature committee (HANG) for the mouse. Asterisks on top of the graph bars indicated the difference between (+Rosi) and (-Rosi) cells was significant after Bonferroni correction for multiple comparison (p <0.0045).

Diabetic patients treated with rosiglitazone showed decreased insulin resistance, lower blood pressure, and increased adiposity [16]–[18]. The higher insulin stimulated 2-deoxyglucose uptake increase in NIH/3T3 adipocytes induced with rosiglitazone suggested that the clinical response might be linked to adipocyte characteristics. The mRNA levels of adipocyte marker genes, genes potentially relevant to blood pressure control, lipid metabolism and glucose uptake in NIH/3T3 adipocytes induced with or without rosiglitazone were compared by qPCR (Figure 6). The NIH/3T3 adipocytes induced without rosiglitazone expressed significantly more angiotensinogen (Agt) and PPARγ (n = 3, p<0.0045).

**Figure 7:**
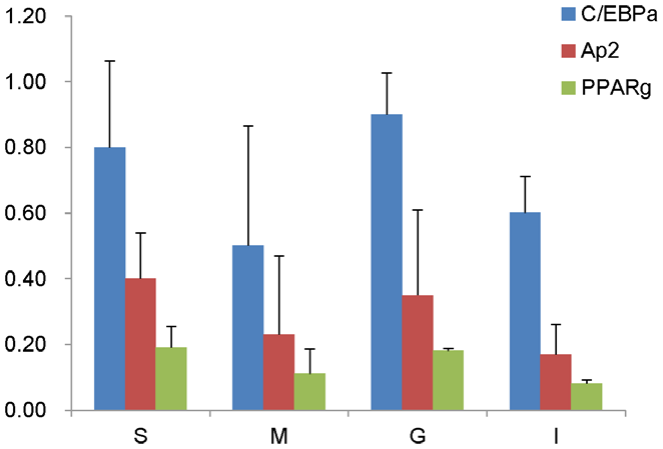
The relative mRNA levels and the standard errors (n = 3) of measurement of Cebpa (C/EBPα), Pparg (PPARγ), Fabp4 (Ap2) genes were determined by qPCR and in adipose tissues taken from 8 weeks old male C57BL/6 mice. G: epididymal fat, M: mesenteric fat, I: interscapular fat, S: posterior thigh fat.

C/EBPα was generally considered an adipocyte marker and an essential gene for adipogenesis. C/EBPα knockout mice lacked adipose tissue. They were very sick and died either *in utero* or soon after birth [19]. Expression of C/EBPβ from the C/EBPα locus in addition to the endogenous C/EBPβ in mice could functionally replace C/EBPα in liver but not in adipose tissue. These mice did develop adipose tissue though the mass was much decreased [20]. To see if adipose tissues without C/EBPα expression exist, the amount of C/EBPα expression in adipose tissues (kindly provided by the center for experimental animals of Tzu Chi University) from C57BL/6 mice was determined by qPCR. We did not find evidence of the existence of adipocytes lacking C/EBPα expression in vivo(Figure 7).

## Discussion

In this work, we tested the hypothesis that increasing the length of induction time during adipogenesis could increase the degree of differentiation to the extent that qualitatively different conclusions would be reached. Our results supported this hypothesis. First we showed that non-confluent 3T3-L1 cells were induced into adipocytes by doubling the induction time or with the help of rosiglitazone. This effect was not limited to 3T3-L1 cells. Multipotential NIH/3T3 is one such cell line that showed increased adipogenicity after prolonged induction. Figure 1 showed that the NIH/3T3 fibroblasts can consistently form insulin responsive adipocytes without exogenous genes when induced with double concentration of FBS for extended period. When testing the adipogenic potential of cell lines or the roles of genes in adipogenesis, the length of induction period above which the cell lines will be judged as non-adipogenic has not been clearly defined. Our results showed that slow rate of adipocytes conversion could be interpreted as inability to form adipocytes in some cases.

Figure 2 showed that the NIH/3T3 cells had barely detectable C/EBPα mRNA and protein levels during adipogenesis. Figure 3 showed that C/EBPα knockdown blocked adipogenesis in 3T3-L1 cells but had no effect in NIH/3T3 cells. Furthermore, whether the knockdown was performed before or after induction of NIH/3T3 cell differentiation did not affect the insulin response of the induced adipocytes. The NIH/3T3 cells were an example of insulin responsive adipocytes formed without C/EBPα.

C/EBPα is a member of the bZIP transcription factor family. Theoretically, the lack of C/EBPα during adipogenesis could be compensated by expression of the other members in this gene family, C/EBPβ and C/EBPδ. This possibility could not be easily tested by inactivating C/EBPβ because of its essential role beyond activating C/EBPα. During 3T3-L1 cells differentiation C/EBPα rose and stayed at near peak level in the adipocytes but C/EBPβ and C/EBPδ peaked early then fell in both 3T3-L1 [21] and NIH/3T3 adipocytes. C/EBPβ fell and rebound slightly and C/EBPδ decreased progressively with time. In addition, when Wu et al. over-expressed C/EBPβ in NIH/3T3 cells to make them inducible like 3T3-L1 cells, continued transcription of C/EBPβ throughout the differentiation process was required. The drop of C/EBPβ level after D2 in Figure 4 did not favor C/EBPβ substituting for C/EBPα in this study, though, this possibility could not be ruled out due to the different induction condition used in that study.

Except for the differences in the expression profiles of the C/EBPs, the insulin signaling patterns of NIH/3T3 and 3T3-L1 adipocytes did not show significant difference. The western blot analysis of the PI3K/Akt pathway in NIH/3T3 shown in Figure 5. The NIH/3T3 adipocytes are a suitable and easier to work with than the 3T3-L1 system to study insulin action. Body weight gain is associated with development of insulin resistance and higher blood pressure. It was seemingly paradoxical that diabetic patients treated with rosiglitazone gained even more weight but showed less insulin resistance and lower blood pressure [22]. The results presented above may provide clues to solve the paradox between obesity and insulin resistance. Figure 1 showed that NIH/3T3 adipocytes induced in the presence of rosiglitazone formed adipocytes faster, became more insulin responsive than those induced without it. The gene expression patterns of these adipocytes were compared in Figure 6.Only angiotensinogen (Agt) and PPARγ showed significant difference between these cells. The effects of rosiglitazone on the NIH/3T3 adipocyte model were consistent with the clinical efficacy observed.

Finally, the NIH/3T3 cells are much easier to work with than the 3T3-L1 cells. The adipogenic potential of NIH/3T3 cells is robust. The same batch has been used for more than 5 years without any noticeable change in differentiation provided they are not - passed continuously in the culture dish. We did not find adipose tissues *in vivo* that express as little C/EBPα as the NIH/3T3 cells (Figure 7), so the significance of this C/EBPα independent pathway in normal adipocyte physiology is unclear. Identification of the alternative signals to turn on the target genes of C/EBPα may someday provide clues to new diabetes treatment.

## Materials and Methods

### Materials

2-Deoxy-D-[2,6-^3^H]glucose was purchased from Amersham Pharmacia Biotech (Piscataway, NJ). Rosiglitazone was purchased from Cayman Chemical (Ann Arbor, MI). Akt and phosphoAkt(pS473) antibodies were from Epitomics (Burlingame, CA). IRS-1 antibody was from Upstate (Charlottesville, VA), and 4G10 was from Millipore (Billerica, MA). All other antibodies were from Genetex (Irvine, CA). The RNeasy total RNA kit was from QIAGEN (Valencia, CA). Reverse transcription reagents were obtained from Promega Corp. (Madison, WI) and TaqMan reagents were from PE Applied Biosystems (Foster City, CA). All other reagents were from Sigma (St. Louis, MO).

### Tissue Culture

3T3-L1 fibroblasts (ATCC, Manassas, VA) were maintained at no higher than 70% confluence in DMEM (Dulbecco's Modified Eagle Medium)/CS (DMEM containing 10% calf serum, 25 mM glucose, 2 mM glutamine). For differentiation they were grown 2 days post confluence in the same medium and then for 3 days in 3T3-L1 induction cocktail (DMEM containing 10% fetal bovine serum, 25 mM glucose, 2 mM glutamine, supplemented with 1 µM dexamethasone, and 0.5 mM isobutylmethylxanthine). The medium was then changed to DMEM/FBS (DMEM containing 10% fetal bovine serum, 25 mM glucose, 2 mM glutamine) for 6 days (change medium every 48 hours) before use. NIH/3T3 fibroblasts (ATCC, Manassas, VA) were maintained at no higher than 70% confluence in DMEM/CS. For differentiation they were grown 2 days post confluence in the same medium and then for 7 days in an adipogenic cocktail containing rosiglitazone (DMEM containing 20% fetal bovine serum, 25 mM glucose, 2 mM glutamine, supplemented with 1 µM dexamethasone, 0.5 mM isobutylmethylxanthine, and 4.5 µM rosiglitazone) or 14 days in an adipogenic cocktail without rosiglitazone. The medium was then changed to DMEM/FBS for 8 days (change medium on every 72 hours) before use.

### Glucose Uptake

Glucose uptake was measured as described previously [23]. Unless otherwise stated, the rates were measured in differentiated 3T3-L1 cells on 9^th^ day post-induction, in NIH/3T3 adipocytes on 15^th^ day post-induction with rosiglitazone or on 22^nd^ day without rosiglitazone, in 12 (or 24)-well plates.

### Oil Red O Staining

3T3-L1 and NIH/3T3 adipocytes in 12 well plates were washed with PBS 2 times, fixed in 3.7% formaldehyde for 2 minutes, washed with ddH2O, then stained in 1 ml of 0.3% Oil Red O (mixture of 40 ml ddH2O and 60 ml 0.5 g oil red o in isopropanol) for 1 hour, then washed with ddH2O twice. Differentiation efficiency was assessed macroscopically using a Sony cybershot T-900 and microscopically using a Nikon COOLPIX 4500 camera mounted on a ZeissAxio VertA1 microscope.

### Western blot analysis

Cells were lysed in a buffer containing 20 mM HEPES, 0.2 M NaCl, 0.5% Triton X-100, 20% glycerol, 1 mM EDTA, 1 mM EGTA, protease and phosphatase inhibitors (Roche Applied Science). After rotating for 30 min at 4°C, cell lysates were centrifuged at 12,000× g for 10 min at 4°C to remove insoluble material. The samples were subjected to sodium dodecyl sulfate-polyacrylamide gel electrophoresis (SDS-PAGE), transferred to a nitrocellulose membrane and probed with the indicated antibodies. The proteins were visualized by enhanced chemiluminescence (Millipore, Temecula, CA) or BCIP/NBT 1-Component Phosphatase Substrate (KPL, Gaithersburg, MD). The intensitiy of each band was quantified by densitometry using the ImageJ software [24].

### RNA Extraction/Quantitative RT-PCR

RNA was extracted from the cells following the manufacturer's instructions. Real time quantitative PCR was performed using an ABI-PRISM 7700 Sequence Detection System instrument and software (PE Applied Biosystems, Inc., Foster City, CA) as described previously. The primers from 5′-3′ for each gene were as follows (gene name, forward; reverse,): Actin, GCATGCAGAAGGAGATCACA, TTGTCGATTGTCGTCCTGAG; Gapdh, AATGTGTCCGTCGTGGATCT; CCCTGTTGCTGTAGCCGTAT; Agt, CACCCCTGCTACAGTCCATT, GTCTGTACTGACCCCCTCCA; Scarb1, CAGGCTGTGGGAACTCTAGC, GAAAAAGCGCCAGATACAGC; Cfd, TGCACAGCTCCGTGTACTTC, CACCTGCACAGAGTCGTCAT; Adipoq, GTTGCAAGCTCTCCTGTTCC, TCTCCAGGAGTGCCATCTCT; Lipe, AGACACCAGCCAACGGATAC, ATCACCCTCGAAGAAGAGCA; Pnpla2, TCCGAGAGATGTGCAAACAG, CTCCAGCGGCAGAGTATAGG; Slc2a4 (Glut4), ACTCTTGCCACACAGGCTCT, AATGGAGACTGATGCGCTCT; Cd36, GAGCAACTGGTGGATGGTTT, GCAGAATCAAGGGAGAGCAC; Cebpa (C/EBPα), TTACAACAGGCCAGGTTTCC, CTCTGGGATGGATCGATTGT; Pparg (PPARγ), TTTTCAAGGGTGCCAGTTTC, AATCCTTGGCCCTCTGAGAT; Fabp4, TCACCTGGAAGACAGCTCCT, AATCCCCATTTACGCTGATG. Cebpb (C/EBPβ), CAAGCTGAGCGACGAGTACA, AGCTGCTCCACCTTCTTCTG; Cebpd (C/EBPδ), ATCGCTGCAGCTTCCTATGT, AGTCATGCTTTCCCGTGTTC.

Results were normalized to the endogenous Gapdh or actin.

### Statistical Analysis

Data are presented as mean ± SE. Statistical significance of treatments was determined by the t test using the Stata 8 software (Texas, USA). P <0.05 was considered statistically significant. The results in Figure 5 were first analyzed by oneway ANOVA and Bartlett's test for equal variance. Bonferroni correction was used in the post hoc analysis for multiple comparisons. Because the amounts of proteins were compared only between the adipocytes that were both treated without or both with insulin, p<0.025 was considered statistically significant after the correction.

### Lentivirus

#### Production

The preparation of transfection quality DNA for the TRC library and production of viruses was done following the protocols of TRC at http://rnai.genmed.sinica.edu.tw/en/Protocols.asp.

#### Infection

Lentivirus infection was done following the protocols of TRC at http://rnai.genmed.sinica.edu.tw/en/Protocols.asp.

The lentiviruses expressing shRNA targeting C/EBPα were RNAi consortium clone ID TRCN0000009502 (C1) and TRCN0000009504 (C3). Those targeting PPARγ were RNAi consortium clone ID TRCN0000001658 (P3), TRCN0000001660 (P5). The construct targeting luciferase was used as the control, Luc(TRCN0000072266).

#### Animals

To avoid unnecessary sacrifice of animals, murine adipose tissues were obtained from the control mice of a related project, which had been approved by the Institutional Animal Care and Use Committee, Tzu Chi University. (Approval Number: 98074). C57BL/6 mice were purchased from the National Laboratory Animal Center of Taiwan. Tissues were isolated from CO2 euthanized animals by the veterinarian of the animal facility.

## References

[1] Rosen ED, MacDougald OA (2006) Adipocyte differentiation from the inside out. Nat Rev Mol Cell Biol 7: 885–896. 17139329 10.1038/nrm2066

[2] Green H, Kehinde O (1975) An established preadipose cell line and its differentiation in culture. II. Factors affecting the adipose conversion. Cell 5: 19–27. 165899 10.1016/0092-8674(75)90087-2

[3] Green H, Meuth M (1974) An established pre-adipose cell line and its differentiation in culture. Cell 3: 127–133. 4426090 10.1016/0092-8674(74)90116-0

[4] Rubin CS, Hirsch A, Fung C, Rosen OM (1978) Development of hormone receptors and hormonal responsiveness in vitro. Insulin receptors and insulin sensitivity in the preadipocyte and adipocyte forms of 3T3-L1 cells. J Biol Chem 253: 7570–7578. 81205

[5] Jainchill JL, Aaronson SA, Todaro GJ (1969) Murine sarcoma and leukemia viruses: assay using clonal lines of contact-inhibited mouse cells. J Virol 4: 549–553. 4311790 10.1128/jvi.4.5.549-553.1969 PMC375908

[6] El-Jack AK, Hamm JK, Pilch PF, Farmer SR (1999) Reconstitution of insulin-sensitive glucose transport in fibroblasts requires expression of both PPARgamma and C/EBPalpha. J Biol Chem 274: 7946–7951. 10075691 10.1074/jbc.274.12.7946

[7] Wu Z, Rosen ED, Brun R, Hauser S, Adelmant G, et al (1999) Cross-regulation of C/EBP alpha and PPAR gamma controls the transcriptional pathway of adipogenesis and insulin sensitivity. Mol Cell 3: 151–158. 10078198 10.1016/s1097-2765(00)80306-8

[8] Rosen ED, Hsu CH, Wang X, Sakai S, Freeman MW, et al (2002) C/EBPalpha induces adipogenesis through PPARgamma: a unified pathway. Genes Dev 16: 22–26. 11782441 10.1101/gad.948702 PMC155311

[9] Lehmann JM, Moore LB, Smith-Oliver TA, Wilkison WO, Willson TM, et al (1995) An antidiabetic thiazolidinedione is a high affinity ligand for peroxisome proliferator-activated receptor gamma (PPAR gamma). J Biol Chem 270: 12953–12956. 7768881 10.1074/jbc.270.22.12953

[10] Lambe KG, Tugwood JD (1996) A human peroxisome-proliferator-activated receptor-gamma is activated by inducers of adipogenesis, including thiazolidinedione drugs. Eur J Biochem 239: 1–7. 8706692 10.1111/j.1432-1033.1996.0001u.x

[11] Guo W, Zhang KM, Tu K, Li YX, Zhu L, et al (2009) Adipogenesis licensing and execution are disparately linked to cell proliferation. Cell Res 19: 216–223. 19065151 10.1038/cr.2008.319

[12] Wang M, Bronte V, Chen PW, Gritz L, Panicali D, et al (1995) Active immunotherapy of cancer with a nonreplicating recombinant fowlpox virus encoding a model tumor-associated antigen. J Immunol 154: 4685–4692. 7722321 PMC1976248

[13] Kaneko T, LePage GA (1978) Growth characteristics and drug responses of a murine lung carcinoma in vitro and in vivo. Cancer Res 38: 2084–2090. 418873

[14] Yeh WC, Cao Z, Classon M, McKnight SL (1995) Cascade regulation of terminal adipocyte differentiation by three members of the C/EBP family of leucine zipper proteins. Genes Dev 9: 168–181. 7531665 10.1101/gad.9.2.168

[15] Payne VA, Au WS, Gray SL, Nora ED, Rahman SM, et al (2007) Sequential regulation of diacylglycerol acyltransferase 2 expression by CAAT/enhancer-binding protein beta (C/EBPbeta) and C/EBPalpha during adipogenesis. J Biol Chem 282: 21005–21014. 17504763 10.1074/jbc.M702871200 PMC2254492

[16] Yki-Jarvinen H (2004) Thiazolidinediones. N Engl J Med 351: 1106–1118. 15356308 10.1056/NEJMra041001

[17] Raji A, Seely EW, Bekins SA, Williams GH, Simonson DC (2003) Rosiglitazone improves insulin sensitivity and lowers blood pressure in hypertensive patients. Diabetes Care 26: 172–178. 12502676 10.2337/diacare.26.1.172

[18] Sarafidis PA, Lasaridis AN, Nilsson PM, Pagkalos EM, Hitoglou-Makedou AD, et al (2004) Ambulatory blood pressure reduction after rosiglitazone treatment in patients with type 2 diabetes and hypertension correlates with insulin sensitivity increase. J Hypertens 22: 1769–1777. 15311106 10.1097/00004872-200409000-00022

[19] Wang ND, Finegold MJ, Bradley A, Ou CN, Abdelsayed SV, et al (1995) Impaired energy homeostasis in C/EBP alpha knockout mice. Science 269: 1108–1112. 7652557 10.1126/science.7652557

[20] Chen SS, Chen JF, Johnson PF, Muppala V, Lee YH (2000) C/EBPbeta, when expressed from the C/ebpalpha gene locus, can functionally replace C/EBPalpha in liver but not in adipose tissue. Mol Cell Biol 20: 7292–7299. 10982846 10.1128/mcb.20.19.7292-7299.2000 PMC86283

[21] Darlington GJ, Ross SE, MacDougald OA (1998) The role of C/EBP genes in adipocyte differentiation. J Biol Chem 273: 30057–30060. 9804754 10.1074/jbc.273.46.30057

[22] Bakris G, Viberti G, Weston WM, Heise M, Porter LE, et al (2003) Rosiglitazone reduces urinary albumin excretion in type II diabetes. J Hum Hypertens 17: 7–12. 12571611 10.1038/sj.jhh.1001444

[23] Stephens JM, Lee J, Pilch PF (1997) Tumor necrosis factor-alpha-induced insulin resistance in 3T3-L1 adipocytes is accompanied by a loss of insulin receptor substrate-1 and GLUT4 expression without a loss of insulin receptor-mediated signal transduction. J Biol Chem 272: 971–976. 8995390 10.1074/jbc.272.2.971

[24] Schneider CA, Rasband WS, Eliceiri KW (2012) NIH Image to ImageJ: 25 years of image analysis. Nat Methods 9: 671–675. 22930834 10.1038/nmeth.2089 PMC5554542

